# Machine learning early prediction of respiratory syncytial virus in pediatric hospitalized patients

**DOI:** 10.3389/fped.2022.886212

**Published:** 2022-08-04

**Authors:** Chak Foon Tso, Carson Lam, Jacob Calvert, Qingqing Mao

**Affiliations:** ^1^Dascena, Inc., Houston, TX, United States; ^2^Montera Inc., San Francisco, CA, United States

**Keywords:** respiratory syncytial virus, pediatric infection, diagnosis, machine learning, algorithm, XGBoost

## Abstract

Respiratory syncytial virus (RSV) causes millions of infections among children in the US each year and can cause severe disease or death. Infections that are not promptly detected can cause outbreaks that put other hospitalized patients at risk. No tools besides diagnostic testing are available to rapidly and reliably predict RSV infections among hospitalized patients. We conducted a retrospective study from pediatric electronic health record (EHR) data and built a machine learning model to predict whether a patient will test positive to RSV by nucleic acid amplification test during their stay. Our model demonstrated excellent discrimination with an area under the receiver-operating curve of 0.919, a sensitivity of 0.802, and specificity of 0.876. Our model can help clinicians identify patients who may have RSV infections rapidly and cost-effectively. Successfully integrating this model into routine pediatric inpatient care may assist efforts in patient care and infection control.

## Introduction

Respiratory syncytial virus (RSV) is the most common lower respiratory tract infection in children; nearly all children have been infected by the time they reach 2 years of age (1). RSV causes mild infection in most healthy children, with symptoms often including fever, nasal congestion, and mild cough (1). However, RSV may also result in severe illness requiring hospitalization. Current estimates suggest that nearly 60,000 children under 5 years of age are hospitalized with RSV annually in the United States (2). Hospitalization rates are high among infants < 6 months old, particularly for those born prematurely (3). The Centers for Disease Control and Prevention (CDC) estimates that 1–2% of RSV infections in this age group result in hospitalization (2, 4). Other risk factors include premature birth, chronic pulmonary or congenital heart disease, immunodeficiencies, or neuromuscular disorders (1, 2). Pediatric patients hospitalized with RSV may require intensive care unit (ICU) admission and mechanical ventilation, which is associated with substantial healthcare spending both during and following treatment (5).

RSV can be detected in infected children through polymerase chain reaction (PCR) testing and, less accurately, through rapid antigen testing (6, 7). Because RSV infections are extremely common in children, current guidelines from the American Academy of Pediatrics recommend against routine screening for RSV in young children presenting with respiratory infection (8), noting that a positive RSV test generally does not change the course of care for patients whose infection can be managed in an outpatient setting. However, RSV testing can be informative for patients treated in hospital settings, where it may help to identify infected patients in need of isolation to prevent outbreaks, as well as identify vulnerable patients in need of additional monitoring and supportive management (8).

The utility of machine learning algorithms (MLAs) to discriminate between COVID-19 and other viral lower respiratory infections in pediatric patients has been established in previous research (9). To guide appropriate use of RSV testing, we have developed a MLA to identify pediatric patients who have RSV upon hospital admission. We present a clinically useful MLA that uses individualized demographics and vital signs data that are routinely collected early upon hospital admission. Infections are substantially enriched among patients identified as high-risk for RSV by our MLA, which demonstrates its utility as a rapid screening tool to help clinicians more efficiently target patients for confirmatory testing and response.

## Materials and methods

### Dataset

In this study, a large commercially available electronic health record (EHR) database was used that collects data from over 700 inpatient and ambulatory care sites located in the United States. Clinical, claims, and other medical administrative data are included in the database. Data was obtained from all emergency department and inpatient encounters for the year 2019. Inclusion criteria included children aged five or younger with at least one measurement of all the required data inputs present within the first 2 h of hospital admission. Patient data was de-identified in compliance with the Health Insurance Portability and Accountability Act and thus, does not constitute human subjects research.

### Data processing

For each patient encounter, only measurements available within the first 2 h of admission were used as inputs to predict RSV positivity. The model used the following inputs, which were all required for the model to make a prediction: age, sex, systolic blood pressure (SysABP), diastolic blood pressure (DiasABP), heart rate (HR), respiratory rate (RespRate), body temperature (Temp), peripheral oxygen saturation (SpO2), height, and weight. Time varying features (e.g., clinical measurements) were summarized by the first, mean, and last measurement within the first 2 h of admission; those three summary statistics were used as features in the model. An 80/20 train/test split *via* randomization was used.

### Gold standard

A positive result of RSV nucleic acid amplification tests (NAATs) such as a PCR test, either from a stand-alone test or as part of a respiratory disease panel, was considered the positive label for our model. NAATs are considered the clinical gold standard for diagnosis of RSV (8, 10). All other RSV tests, such as antigen tests, were disregarded by the model. As the model presented here is a binary classification model, all non-positive encounters were automatically considered negative.

### Cohort definition

**FIGURE 1:**
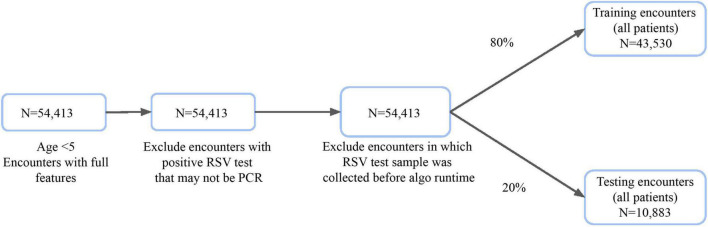
Inclusion criteria for training and testing datasets of patient hospital encounters for algorithm development.

The attrition process for the MLA is illustrated in Figure 1. We excluded some patients who had a positive RSV test result, as we could not conclusively determine that it was resulting from an NAAT. We also excluded patients whose RSV test samples were collected within the first 2 h after admission. The final population consisted of 54,413 patients who were randomly split into train and test sets.

### Machine learning nodel

We used XGBoost (XGB, or extreme gradient boost), a class of gradient boosted decision tree implemented it using the XGBoost library in Python (11, 12). We took advantage of the versatility of the algorithm as XGB is highly interpretable and performs well for an imbalanced dataset (12). A grid search cross-validation was performed to determine the optimal parameters. The parameters used in the final model are reported in Supplementary Table 1.

### Statistical analysis

95% confidence intervals (CIs) were reported for model performance. For the area under the receiver-operating curve (AUROC), bootstrap sampling with replacement of prediction indices was used to generate multiple receiver-operating curves (ROC) and the area under each curve was calculated. We then reported the 5th and 95th percentile values of AUROC. For other performance metrics, 95% CIs were calculated using normal approximation. For the demographics table, Fisher’s Exact tests were performed between the positive and negative groups to obtain *p*-values.

## Results

To develop and test our models, we used hospital records for 54,413 encounters with patients aged 5 years or younger. Prior to algorithm trigger time, no RSV diagnostic tests had been documented and no RSV tests had been performed within 2 h of admission (Figure 1). These encounters were divided into a training set with 80% (*n* = 43,530) and a holdout test set with 20% (*n* = 10,883) of encounters. We observed demographic differences in age between encounters with and without positive RSV tests (Table 1). RSV-positive encounters had higher proportions of patients aged 1–3 years (*p* < 0.001); encounters without positive RSV tests had higher proportions of patients who were aged less than 1 year (*p* = 0.002) or aged 4–5 years (*p* < 0.001) or preterm birth (*p* < 0.001). The prevalence of RSV in the holdout test set, as measured by NAAT, was 1.8% (*n* = 197 RSV-positive), with a test positivity rate of 18.7%.

**TABLE 1:** Demographic data of non-RSV positive and RSV positive patients with hospital encounters included in the holdout test set.

Demographics	Training set (*N* = 43,530)	Testing set		
		Non-RSV positive (*N* = 10,686)	RSV positive (*N* = 197)	*P*-value
Below 1 years old	21,204 (48.7%)	5,244 (49.1%)	61 (31.0%)	0.002
1–3	12,552 (28.8%)	3,024 (28.3%)	120 (60.9%)	*p* < 0.001
4–5	9,772 (22.4%)	2,418 (22.6%)	16 (8.1%)	*p* < 0.001
Unknown age	2 (0.0%)	0 (0.0%)	0 (0.0%)	1
Male	2,363 (54.3%)	5,791 (54.2%)	107 (54.3%)	1
Female	19,743 (45.4%)	4,860 (45.5%)	90 (45.7%)	1
Unknown sex	153 (0.4%)	35 (0.3%)	0 (0.0%)	1
White	24,065 (55.3%)	5,985 (56.0%)	118 (59.9%)	0.594
Hispanic	5,184 (11.9%)	1,234 (11.5%)	23 (11.7%)	0.911
Black	5,997 (13.8%)	1,441 (13.5%)	21 (10.7%)	0.342
Asian	1,142 (2.6%)	257 (2.4%)	8 (4.1%)	0.158
Other/unknown	7,142 (16.4%)	1,769 (16.6%)	27 (13.7%)	0.439
Preterm birth	2,786 (6.4%)	713 (6.7%)	1 (0.5%)	*p* < 0.001
Smoking exposure	240 (0.6%)	78 (0.7%)	0 (0.0%)	0.407
Congenital heart defects	485 (1.1%)	140 (1.3%)	0 (0.0%)	0.185
Neuromuscular disorders	7 (0.0%)	1 (0.0%)	0 (0.0%)	1
Down syndrome	77 (0.2%)	20 (0.2%)	1 (0.5%)	0.320
Cystic fibrosis	11 (0.0%)	7 (0.1%)	1 (0.5%)	0.137
Chronic lung disease	266 (0.6%)	80 (0.7%)	1 (0.5%)	1
Pediatric immunodeficiency	70 (0.2%)	15 (0.1%)	0 (0.0%)	1
RSV PCR test performed	4,175 (9.6%)	851 (8.0%)	197 (100.0%)	*p* < 0.001
RSV PCR test positive	719 (1.7%)	0 (0.0%)	197 (100.0%)	*p* < 0.001

**FIGURE 2:**
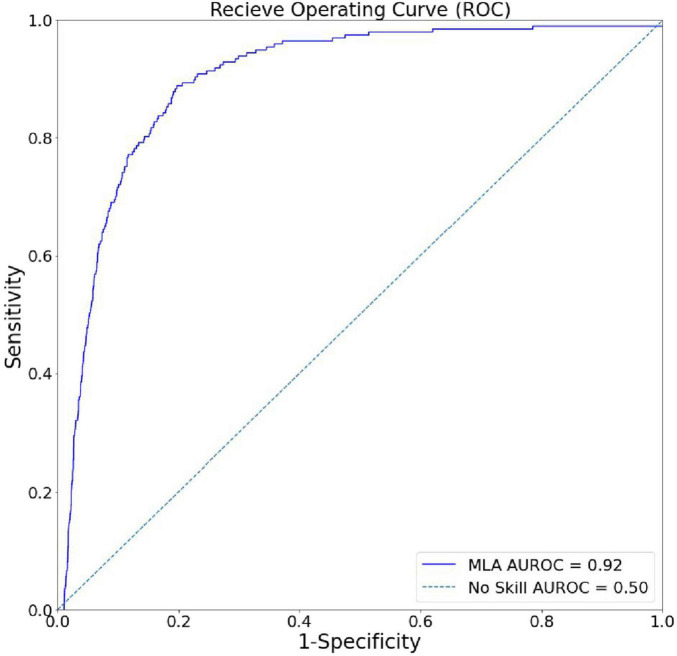
Algorithm discrimination and precision in identifying hospital encounters with future positive RSV tests. The receiver-operating curve (ROC) for the XGBoost model, showing superiority to random chance (gray) in discrimination between RSV-positive and non-RSV-positive encounters.

Figure 2 shows the ROC of the XGBoost model. The AUROC for our model was 0.919, demonstrating exceptionally high accuracy in distinguishing RSV-positive encounters as positive and non-RSV-positive encounters as non-positive in a binary classification task. Fixing the sensitivity of the model to 0.80 yielded a specificity of 0.876 (Table 2).

**TABLE 2:** Summary of algorithm performance metrics.

Performance metric	Value (95% CI)
AUROC	0.919 (0.906–0.932)
Sensitivity	0.802 (0.746–0.858)
Specificity	0.876 (0.87–0.882)

AUROC, area under the receiver–operating curve (no-skill baseline = 0.50). Optimal specificity was determined with a minimal sensitivity of 0.8.

**FIGURE 3:**
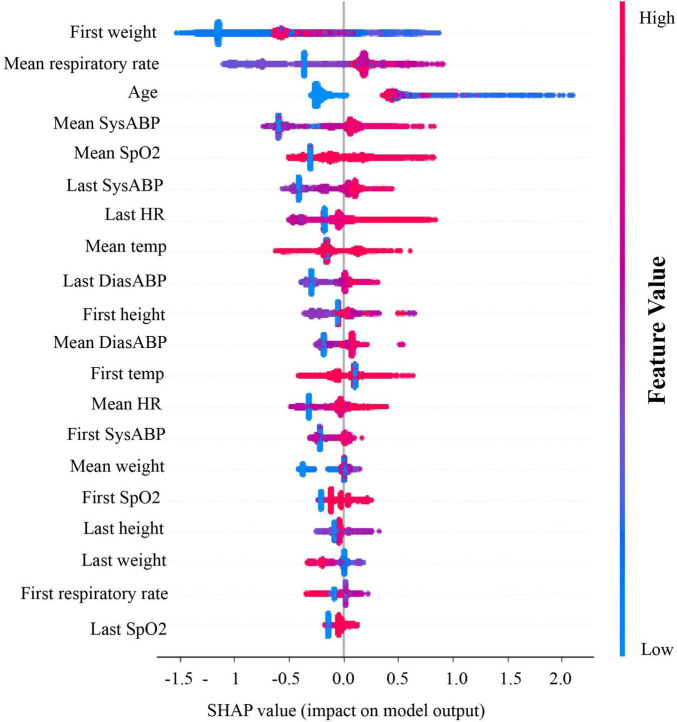
Shapley value plots for degree of model’s dependence on specific features. From top to bottom, the relative importance of each feature was ranked. Red dots represent relatively high values of a feature and blue dots represent relatively low values. On the x-axis, the SHAP values (or impact on model output) is plotted. If most of the red dots are on the right of the x-axis, it means high value of that feature (ex. mean DiasABP in this figure) substantially contributes to a positive prediction. SysABP, systolic arterial blood pressure; DiasABP, diastolic arterial blood pressure; RespRate, respiratory rate; HR, heart rate; SpO2, oxygen saturation; Temp, body temperature.

To determine which features of patient encounters most strongly influenced our model’s prediction of RSV, we generated summary plots from Shapley additive explanations (SHAP) analyses (Figure 3) (13). Our model showed a strong dependence on patient weight and age, together with systolic or diastolic blood pressure and high respiratory rate (14). These results show that our model’s ability to successfully distinguish future RSV positivity among hospitalized pediatric patients is most strongly dependent on vital signs and clinical data that are routinely and rapidly collected at patient point-of-care.

## Discussion

In this study, we developed an MLA to rapidly and systematically predict a positive RSV NAAT test among hospitalized pediatric patients. This algorithm used inputs that are routinely collected and reported in patients’ EHRs within 2 h of admission to predict a positive NAAT for RSV later in the same admission. Our work demonstrates the utility of leveraging machine learning techniques to rapidly predict previously unidentified infections among hospitalized patients.

There are two major innovations in our study that substantially contribute to the field. First, our study focuses specifically on identifying likely RSV infections rapidly upon presentation to a hospital emergency room. This differs from previously developed MLAs focused on pediatric RSV infections, which have focused either on predicting future RSV diagnosis, hospitalization, or severe progression of disease in the months to years following data collection, or on identifying RSV infections among pediatric patients that were already hospitalized with known symptoms of respiratory viral infection (15–17). Several of these previous algorithms also were developed using data only from preterm infants (16, 17), thereby limiting their generalizability as compared to our MLA. As a preventive tool, Heaton et al. developed an MLA to predict seasonal RSV outbreaks to allow for timely immunoprophylaxis injections for children predisposed to poor infection outcomes (18). Other studies using MLAs that predict suitable treatment courses (19) or patient outcomes (15, 20) for bronchiolitis patients, a disease commonly caused by RSV, require a proper diagnosis prior to running the algorithm. These RSV preventive and treatment studies do not address the need for broad screening of incoming pediatric patients and rapid identification of RSV infected patients. Our study therefore provides unprecedented utility among RSV-focused MLAs for hospital healthcare providers to improve the efficiency and accuracy of their initial care for pediatric patients. Second, our MLA is designed to predict RSV positive tests without requiring detailed patient data that require surplus time and effort over standard-of-care protocols performed early in hospitalization. This differs from previously developed risk scores or MLAs that required inputs of ICD diagnosis codes, transcriptome data, and/or documentation of specific symptoms that take additional time to collect and log in patients’ EHRs (16, 17, 21–23). The relative simplicity of our MLA indicates that integration into hospital settings would be more efficient and immediately useful to clinicians who care for pediatric inpatients.

If successfully implemented as a rapid, preliminary RSV screening system in a hospital setting, our algorithm could provide several primary services to healthcare providers caring for pediatric patients. First, it could be used as a tool for identifying patients to be enrolled or not enrolled in cohort studies or clinical trials that involve active RSV infection - either to include or exclude patients who are actively infected (24). This would save clinical researchers time and effort by substantially narrowing their scope of viral testing. Second, it could help hospital infection prevention personnel to more quickly identify infected patients who may need to be placed on additional precautions to prevent healthcare-associated transmission of RSV. Outbreaks of RSV in pediatric hospital settings are well documented and have been shown to contribute to increased patient morbidity, mortality, and complexity of care (6, 25, 26). Third, our algorithm could better inform delivery of care for infected patients by identifying them more rapidly and with greater efficiency of viral testing. Taken together, these advantages could be leveraged particularly well in tertiary care research and teaching hospitals that would benefit from an efficient alternative to established risk scores or systematic viral testing to identify infected patients.

There are several limitations to this study. First, the use of NAAT testing for RSV as a “gold standard” likely excluded many diagnoses of infection by rapid antigen detection, which may have skewed the RSV prevalence and predictive power of the MLA. Second, we did not include data on the presence or absence of respiratory symptoms that are known to be strong predictors of RSV infection (2, 8, 27), because these data were often missing from EHRs of the patients included in this study. Future directions of this research could potentially be improved by considering RSV diagnoses made by rapid antigen testing. Additionally, future studies should include the presence or absence of known symptoms of acute respiratory disease to identify patients with RSV.

## Conclusion

The model we present in this study performed well in identifying RSV infections among pediatric inpatients at the time they presented to the hospital, using clinical data that are routinely collected in the first 2 h following admission. Our model demonstrates utility for clinicians who would benefit from rapidly identifying RSV infections among pediatric inpatients for purposes of infection prevention, clinical trial enrollment, or management of care. Future directions in the field include refining diagnostic algorithms by including more detailed patient data and the development of new models focused on other infectious diseases of substantial clinical concern.

## Data availability statement

The original contributions presented in this study are included in the article/Supplementary material, further inquiries can be directed to the corresponding author.

## Ethics statement

This research only used de-identified patient data, thus it is exempt from ethical approval, in line with local legislation.

## Author contributions

All authors listed have made a substantial, direct, and intellectual contribution to the work, and approved it for publication.
