# Supplementary material for: Machine learning early prediction of respiratory syncytial virus in pediatric hospitalized patients
Source: Front Pediatr. 2022 Aug 4;10:886212. doi: 10.3389/fped.2022.886212 (PMC9385995; doi:10.3389/fped.2022.886212)
Supplement: Supplementary file 1 [file Table_1.docx]

**Supplementary Materials**

**Supplementary Table 1. Parameters used in the final model**

| **Parameter** | **Value** |
| --- | --- |
| learning_rate | 0.1 |
| max_depth | 7 |
| n_estimators | 150 |
| reg_lambda | 0.5 |
| scale_pos_weight | 2 |
